# Commercial influences on infant and young child feeding

**DOI:** 10.1016/j.fhj.2025.100264

**Published:** 2025-06-30

**Authors:** Bartosz Helfer, Katarzyna Henke-Ciążyńska, Robert J. Boyle

**Affiliations:** a Meta-Research Centre, University of Wrocław, Wrocław, Poland; b Institute of Psychology, University of Wrocław, Wrocław, Poland; c School of Public Health, Imperial College London, London, UK

## Abstract

Optimal infant and young child feeding is essential for child health, development and social wellbeing. Breastfeeding is the foundation of early nutrition, and powerfully protects infants and young children against both infectious and non-communicable diseases. The commercial milk formula industry systematically undermines breastfeeding through pervasive marketing and influence over science, education and policy, despite international guidance, including the World Health Organization (WHO) International Code of Marketing of Breast-milk Substitutes. Biased industry-sponsored research supports misleading claims about formula efficacy, while industry funding of nutrition regulators, professional education and healthcare practitioners creates conflicts of interest. To protect public health, stronger regulations, full implementation of the WHO Code and clear boundaries between the formula industry and health sectors are needed. We propose policy actions, including a binding global treaty and transparency measures, to counteract commercial influence and safeguard child health.

Optimal infant and young child feeding (IYCF) underpins child survival, growth and cognitive development, while promoting bonding and social integration.^1^ Scaling up breastfeeding to near-universal levels could avert over 800,000 child deaths annually and prevent hundreds of billions in economic losses through improved health and development.^2^ Breastfeeding is associated with long-term protection against non-communicable diseases, including improved metabolic and cardiovascular outcomes. As part of this protective effect, it contributes to lower risk of overweight and obesity – an especially relevant benefit in the context of the escalating global obesity crisis.^3^

IYCF transcends nutrition, shaping relationships, family structures, economic development and social cohesion. Feeding is an intimate act that reinforces the parent–child bond and engages support networks, making IYCF a collective societal responsibility.^4^ The way that societies enable or impede healthy IYCF practices intersects with gender equity (eg through maternity protections), social justice and long-term human capital development.^5^

Since the late 1800s, the commercial milk formula (CMF) industry has played an increasing role in influencing IYCF, following the mass marketing of the first substitutes for human milk.^6^ Early artificial feeding was associated with elevated risks of morbidity and mortality, primarily due to microbial contamination and milk spoilage.^6^ In the 20th century, aggressive formula promotion contributed to a global decline in breastfeeding and documented increases in infant morbidity and mortality.^6^ This historic pattern of commercial influence undermining child health continues today. Modern CMF marketing – now a US$55 billion global industry – continues to undermine breastfeeding, driving avoidable mortality and morbidity among infants and young children.^7^ The CMF industry’s marketing strategies – broad in scope and embedded across systems – pose a major long-term public health challenge as a commercial determinant of child health.

The CMF industry’s marketing strategies extend beyond direct-to-consumer promotion, encompassing influence on key systemic domains, including science, professional education and regulatory processes (Fig. 1). Below, we examine these mechanisms and propose solutions to mitigate their impact, drawing on global evidence and World Health Organization (WHO) guidance.

**Fig. 1:**
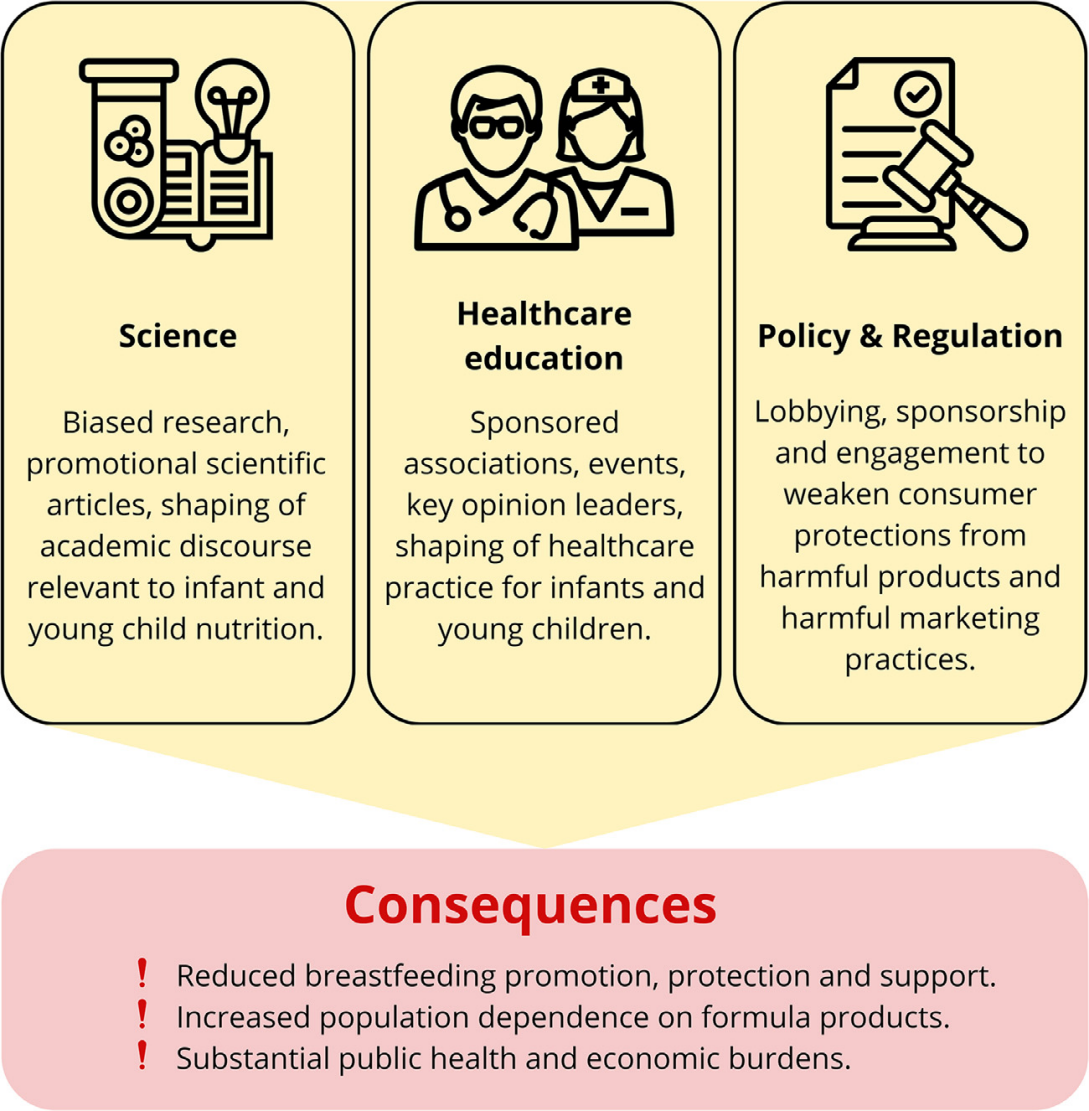
Key domains of commercial milk formula industry influence.

One key domain of commercial influence is science. A recent commentary highlighted how a major scientific journal was used by a CMF company to publish what were essentially marketing materials masquerading as research.^8^ When journals accept industry funding – whether in the form of sponsored supplements, advertisements, or even article-processing charges paid by CMF manufacturers – they enter into a conflict of interest (COI) that can potentially erode scientific integrity and editorial independence. The practice has drawn sharp criticism and calls have been made for journals to cease accepting money from CMF manufacturers altogether in order to protect the objectivity of science.^9^^,^^10^

Accepting industry influence through funding of research, publications and academic conferences means that scientific discourse on IYCF becomes framed by commercial interests.^11^ This industry-driven framing and prioritisation of science can exaggerate the role of CMF or specific additives^8^ and downplay associated harms. Industry messaging can also divert attention and resources away from independent research priorities.^8^^,^^12^

In the realm of education, financial sponsorship of professional events and training by CMF industry is widespread.^13^ For instance, CMF manufacturers commonly fund or host conferences, continuing medical education (CME) seminars, and workshops for healthcare professionals relevant to maternal and child health.^14^^,^^15^ They provide speakers or ‘educational’ materials that promote their products, ingredients and related scientific narratives.^16^

Representatives of CMF companies also access maternity wards and healthcare clinics, where they distribute free samples and promotional pamphlets under the guise of supporting patient education.^16^^,^^17^ These materials often carry logos and messaging that normalise or idealise formula feeding. Only 79 countries (out of 194) prohibit the promotion of CMFs in health facilities, and just 51 ban the distribution of free or low-cost formula supplies within the healthcare system.^18^ Thus, in many regions caregivers receive formula samples or see posters and gift packs provided by CMF industry, directly in health settings where they might expect unbiased advice. Likewise, many paediatric professional associations allow industry sponsorship of their activities.^19^ The result is that a large proportion of healthcare workers worldwide are exposed to CMF marketing in what should be educational environments. The WHO has identified marketing through trusted healthcare professionals as a ‘major barrier’ to improving IYCF outcomes.^18^

Sophisticated mass media advertising and digital marketing on social platforms allow CMF companies to reach parents directly, again often blurring the line between information and advertisement.^5^ They commonly make nutrition claims – for example, suggesting a certain formula ‘helps brain development’ – claims that are not scientifically substantiated^20^ and can prey on parental anxieties, eroding confidence in breastfeeding.^5^

More broadly, there is evidence that the CMF industry exerts political influence on governments through lobbying, legal threats, economic diplomacy, and the use of front groups. In 2006, an industry association took the Philippines government to court to block new CMF marketing restrictions, while also seeking to weaken enforcement through parliamentary lobbying and pressure from international business groups.^21^ At the 2018 World Health Assembly, the United States delegation allegedly threatened trade sanctions against Ecuador to derail a pro-breastfeeding resolution – an example of political pressure aligning with CMF industry aims.^22^ Similarly, in Vietnam, just before a proposed ban on formula advertising was scheduled for a vote, the United States Embassy intervened with a letter claiming that the regulation would inflict significant economic harm, citing concerns raised by United States formula companies.^21^ The industry also operates through trade associations such as Specialised Nutrition Europe, which lobby policymakers and standard-setting bodies, sometimes adopting public-interest-style names while advancing corporate agendas.^23^ There is growing recognition that the CMF industry is adopting tactics similar to those historically employed by the tobacco industry to secure favourable regulatory and political environments.^21^^,^^23^

Many countries still lack fully enforceable regulations on marketing of CMFs. According to the latest WHO monitoring, while 136 out of 194 countries have some legal measure reflecting the International Code of Marketing of Breast-Milk Substitutes (Code), only a minority have robust laws covering all Code provisions, and violations in these countries are common, due to weak monitoring and enforcement.^24^ In some instances, regulations have been rolled back: for example, China had adopted some Code provisions in the 1990s, but in 2017 the government removed the Code from its regulations entirely, with little public justification and, as argued elsewhere, possibly influenced by CMF industry pressure.^25^ As *The Lancet* 2023 breastfeeding series noted, the CMF industry has built a network of unaccountable trade associations and front organisations that work to influence political decisions and regulatory processes Box 1.^5^^,^^7^^,^^26^

**Box 1:** Industry narrative versus evidence. These two examples illustrate the discrepancy between commercial marketing messages and scientific evidence, highlighting concerns about industry-driven research biases and the need for evidence-based regulatory approaches.

**DHA in infant formula**	**Cow’s milk-related symptom score (CoMiSS™)**
Docosahexaenoic acid (DHA), a long-chain polyunsaturated fatty acid, is marketed by the formula industry as enhancing cognitive development. Following these claims, the European Union has mandated the inclusion of DHA in all infant formulas.^27^ However, robust scientific evaluation challenges these commercial assertions.^28^^,^^29^ A recent UK-based data linkage study^28^ acquired individual participant data from seven dormant randomised controlled trials of DHA-supplemented formula versus standard formula and linked to the participants’ long-term cognitive outcomes (academic performance at ages 11 and 16). They found no cognitive benefit at age 16 across any of the trials. In fact, secondary outcomes at age 11 indicated potential adverse effects on academic performance (mathematics and English) in infants randomised to DHA-supplemented formula.	CoMiSS is a symptom awareness tool for cow’s milk allergy developed by child health specialists in 2014 with formula company sponsorship. CoMiSS is widely presented at industry-sponsored symposia and conferences and in scientific publications.^30^ CoMiSS lists common symptoms which are seen in most healthy infants and labels them as abnormal. For example, loose or watery stools, which are almost universal in breastfed infants in the early weeks of life, are suggested by CoMiSS to be a symptom of allergy.^31^ CoMiSS is likely to contribute to milk allergy overdiagnosis,^31^ causing unnecessary exposure of infants to unhealthy, glucose syrup-based specialised formula products, and reducing breastfeeding confidence.

To address these challenges, one essential step is to recognise that the CMF industry’s interests are fundamentally in conflict with public health. Just as the world has come to treat tobacco companies as adversaries to health, and not stakeholders in policymaking, a paradigm shift is needed in how we engage with CMF manufacturers on matters of IYCF. The CMF industry’s strong commercial incentives to maximise shareholder value and profit conflict with the health sector’s commitment to promote, support and protect breastfeeding, appropriate complementary feeding and, when needed, to support safe and affordable formula feeding.^14^ Although CMF companies often promote voluntary corporate responsibility pledges – such as providing ostensibly neutral nutrition education to caregivers – these initiatives cannot erase that inherent conflict. Therefore, governments and health institutions should consider adopting a relationship with the CMF industry, akin to the Framework Convention on Tobacco Control’s Article 5.3.^32^ Practical steps to operationalise this approach are outlined in Box 2.

**Box 2:** Priority actions to protect infant and young child feeding from commercial influence. The proposed actions draw conceptual inspiration from Article 5.3 of the WHO FCTC, which mandates the protection of public health policy from commercial and other vested interests. While some points reflect direct analogues, others extend the logic of Article 5.3 through implementation tools used in other sectors – such as ‘Sunshine Act’. The list was informed by WHO guidance on managing COI in nutrition policy, examples from professional self-regulation, and approaches to professional education on industry influence (see references in Box 2).

**1. Restrict industry involvement in policy and public health**
Exclude CMF companies from public health partnerships, policy dialogues on nutrition, and sponsorship or co-branding of campaigns. Maintain a conflict-of-interest framework that restricts their role to regulatory compliance rather than policy shaping.
**2. Strengthen global governance through a binding treaty**
Establish an international treaty on IYCF, similar to frameworks developed for tobacco control. *The Lancet*’s 2023 breastfeeding series calls for a legally binding instrument to end irresponsible CMF marketing and lobbying, strengthening the voluntary WHO Code into a unified global standard, acknowledging that industry self-regulation has failed.^5^^,^^7^^,^^26^
**3. Treat CMF industry funding as a high-risk conflict of interest and dependency**
Enforce stringent COI policies across health associations, journals and NGOs, treating CMF industry funding as high-risk or disqualifying, akin to tobacco or pharmaceutical funding. Recent examples include the Royal College of Paediatrics and Child Health (UK) and the *BMJ* journals discontinuing support from formula manufacturers.^9^
**4. Legislate the International Code of Marketing of Breast-Milk Substitutes in full**
Fully integrate the Code into national legislation, closing regulatory gaps so that all provisions (and subsequent WHA resolutions, such as WHA 69.9 on ending inappropriate promotion of foods for infants and young children)^33^ have legal force. This includes restricting the marketing of products for children up to 3 years of age and covering online and social media channels.
**5. Implement mandatory transparency mechanisms**
Mandate transparency of industry funding and interactions through a publicly searchable database, following a ‘Sunshine Act’ model.^34^
**6. Remove CMF influence from health professional education**
Incorporate the WHO Code into curricula for healthcare professionals. Accreditation and licensure bodies can reinforce this by embedding Code compliance into ethics codes or standards of practice. Tie CPD/CME credits to industry-free education only. Events sponsored by CMF companies would not accrue credits, mirroring the Baby-Friendly Hospital Initiative principle (Step 1a of the Ten Steps).^35^ Linking accreditation to Code adherence would likely sever industry influence in professional education over time.

In conclusion, CMF industry manipulation of science, healthcare practitioner education and the regulatory environment is both a historic and a current example of commercial influences on health. Despite a voluntary international Code of marketing these products since 1981, egregious marketing practices continue to be widespread, with serious public health consequences. Strengthened international regulation is required to prevent widespread harm to young children and their carers through these practices.

The commercial milk formula industry uses science, healthcare practitioners and policymakers to achieve their marketing aims of undermining breastfeeding, their main competitor, and inflating the market for formula.

## CRediT authorship contribution statement

**Bartosz Helfer:** Writing – review & editing, Writing – original draft, Conceptualization. **Katarzyna Henke-Ciążyńska:** Writing – review & editing, Writing – original draft, Visualization, Conceptualization. **Robert J. Boyle:** Writing – review & editing, Writing – original draft, Project administration, Conceptualization.

## Declaration of competing interest

The authors declare the following financial interests/personal relationships which may be considered as potential competing interests:

Robert Boyle reports a relationship with John Wiley & Sons Inc that includes: employment. Robert Boyle reports a relationship with British Society for Allergy and Clinical Immunology that includes: funding grants. Robert Boyle reports a relationship with World Health Organization that includes: consulting or advisory. Robert Boyle reports a relationship with Norwegian Department of Health that includes: consulting or advisory. Robert Boyle reports a relationship with European Academy of Allergy and Clinical Immunology that includes: travel reimbursement. Robert Boyle reports a relationship with Taus, Cebulash and Landua that includes: paid expert testimony. If there are other authors, they declare that they have no known competing financial interests or personal relationships that could have appeared to influence the work reported in this paper.
